# The Myosin Motor Domain-Containing Chitin Synthases Are Involved in Cell Wall Integrity and Sensitivity to Antifungal Proteins in *Penicillium digitatum*

**DOI:** 10.3389/fmicb.2019.02400

**Published:** 2019-10-18

**Authors:** Mónica Gandía, Sandra Garrigues, Begoña Bolós, Paloma Manzanares, Jose F. Marcos

**Affiliations:** Department of Biotechnology, Instituto de Agroquímica y Tecnología de Alimentos, Consejo Superior de Investigaciones Científicas, Valencia, Spain

**Keywords:** chitin, cell wall, antifungal proteins, conidia, *Penicillium digitatum*, virulence, postharvest

## Abstract

*Penicillium digitatum* is the main postharvest pathogen of citrus fruit and is responsible for important economic losses in spite of the massive use of fungicides. The fungal cell wall (CW) and its specific component chitin are potential targets for the development of new antifungal molecules. Among these are the antifungal peptides and proteins that specifically interact with fungal CW. Chitin is synthesized by a complex family of chitin synthases (Chs), classified into up to eight classes within three divisions. Previously, we obtained and characterized a mutant of *P. digitatum* in the class VII gene (Δ*chsVII*), which contains a short myosin motor-like domain (MMD). In this report, we extend our previous studies to the characterization of mutants in *chsII* and in the gene coding for the other MMD-Chs (*chsV*), and study the role of chitin synthases in the sensitivity of *P. digitatum* to the self-antifungal protein AfpB, and to AfpA obtained from *P. expansum*. The Δ*chsII* mutant showed no significant phenotypic and virulence differences with the wild type strain, except in the production and morphology of the conidia. In contrast, mutants in *chsV* showed a more dramatic phenotype than the previous Δ*chsVII*, with reduced growth and conidial production, increased chitin content, changes in mycelial morphology and a decrease in virulence to citrus fruit. Mutants in *chsVII* were specifically more tolerant than the wild type to nikkomycin Z, an antifungal inhibitor of chitin biosynthesis. Treatment of *P. digitatum* with its own antifungal protein AfpB resulted in an overall reduction in the expression of the chitin synthase genes. The mutants corresponding to MMD chitin synthases exhibited differential sensitivity to the antifungal proteins AfpA and AfpB, Δ*chsVII* being more susceptible than its parental strain and Δ*chsV* being slightly more tolerant despite its reduced growth in liquid broth. Taking these results together, we conclude that the MMD-containing chitin synthases affect cell wall integrity and sensitivity to antifungal proteins in *P. digitatum*.

## Introduction

Fungi are responsible for serious diseases in plants, animals and humans, resulting in a large number of deaths and millionaire economic losses (Brown et al., 2012; Fisher et al., 2012). Effective control of phytopathogenic fungi is a major challenge due to the huge economic losses they cause, the emergence of resistance against the few classes of currently available fungicides and the cross-resistance between clinic and crop pathogens (Perfect, 2016; Fisher et al., 2018). In this context, the study of antifungal peptides and proteins has attracted much attention as promising biomolecules to control deleterious fungi (Marcos et al., 2008; Galgóczy and Marx, 2019).

The fungal cell wall (CW) is a promising target for the development of new antifungal molecules because it has a structure and composition not shared by animal or plant cells, and therefore is specific to fungi (Latgé, 2007; Lee and Sheppard, 2016). The CW is also essential as it protects the cell and interacts firstly with the host during infection and colonization, being considered important in the host-pathogen interaction (Kombrink et al., 2011; Sánchez-Vallet et al., 2014; Lee and Sheppard, 2016; Geoghegan et al., 2017; Latgé et al., 2017). Among the unique structure of the fungal CW stands out chitin, a linear homopolymer of β-(1,4)-*N*-acetylglucosamine neither present in plants nor higher animals, necessary for fungal survival and considered as a pathogen-associated molecular pattern (PAMP) (Kombrink et al., 2011; Thomma et al., 2011). Chitin and chitin synthesis are, therefore, targets to develop new antifungal drugs. Chitin synthesis is a complex process carried out by a repertoire of conserved enzymes known as chitin synthases (Chs) encoded by specific genes (*chs*). Three *chs* genes have been reported in the yeast *Saccharomyces cerevisiae*, whereas many more have been identified in filamentous fungi, as it is the case of the Blastocladiomycete *Allomyces macrogynus* or the Zygomycete *Rhizopus chinensis* with 38 and 44 different *chs*, respectively (Gonçalves et al., 2016; Li et al., 2016). These Chs are classified into three divisions and up to eight classes: division I contains class I, II and III *chs* genes; division II contains class IV, V and VII and finally division III contains class VI (Latgé, 2007; Sheng et al., 2013; Gandía et al., 2014; Li et al., 2016; Liu et al., 2016; Roncero et al., 2016). A new Chs class (VIII) in division I was defined recently (Li et al., 2016). Particularly significant are ChsV and ChsVII, which are two chitin synthases with a chimeric structure containing an N-terminal myosin motor-like domain (MMD) fused to a CHS domain, and which have been associated with hyphal growth and virulence in many filamentous fungi (Takeshita et al., 2006; Kim et al., 2009; Larson et al., 2011; Fernandes et al., 2016). Numerous studies on the biological role of the distinct *chs* genes in fungal pathogens have been conducted, obtaining a repertoire of deletion mutants of these genes in different filamentous fungi such as *Aspergillus fumigatus* (Muszkieta et al., 2014), *Fusarium graminearum* (Cheng et al., 2015), *Magnaporthe oryzae* (Kong et al., 2012) or *Botrytis cinerea* (Morcx et al., 2013). Overall, these studies have shown specific roles for each class/division, including the response to CW stress and involvement in virulence.

*Penicillium digitatum* is the main postharvest pathogen of citrus fruit and causes the green mold disease, being responsible for significant economic losses in citriculture (Marcet-Houben et al., 2012; Palou, 2014; Costa et al., 2019). We have previously identified and analyzed the expression of chitin synthase genes in *P. digitatum* (Gandía et al., 2012, 2014). Gene expression analyses concluded that *chsIII* is the most expressed and *chsII* is the least expressed of all the chitin synthase genes; *chsI*, *chsIII, chsV*, and *chsVII* were induced during fruit infection; and *chsII* was the most upregulated gene during axenic growth in solid PDA medium, coincident with conidia production (Gandía et al., 2012, 2014). Moreover, our group disrupted *chsVII* in *P. digitatum* and showed the importance of this gene for fungal growth, conidia production, virulence, CW integrity and mycelium development during fruit infection (Gandía et al., 2014). In addition, this Δ*chsVII* mutant registered an impressive compensatory induction of *chsII* expression and, to a lesser extent, of *chsV*, compared with the parental wild type, during axenic growth and in response to specific stresses such as osmotic shock caused by sorbitol.

Antifungal proteins (AFPs) produced by filamentous fungi from the class Eurotiomycetes hold great promise for the control of fungal pathogens. AFPs are small, cationic, cysteine-rich proteins that are extraordinarily stable and secreted in high amounts by producing fungal strains (Meyer, 2008; Hegedüs and Marx, 2013; Galgóczy and Marx, 2019). The two most studied AFPs are AFP from *Aspergillus giganteus* (Meyer, 2008) and PAF from *Penicillium chrysogenum* (Marx et al., 2008). Studies in our group have recently identified, produced and characterized two highly active AFPs from postharvest pathogens: AfpB from *P. digitatum* (Garrigues et al., 2016, 2017) and AfpA from *Penicillium expansum* (Garrigues et al., 2018), which are both active even against the self-fungi. Moreover, both AFPs can control experimental infections of *P. digitatum* and *B. cinerea* on citrus fruit and tomato plants, respectively (Garrigues et al., 2018; Shi et al., 2019). We have initiated the study of the mode of action of these AFPs, showing that AfpB induces the phosphorylation of mitogen-activated protein kinases (MAPKs), including Slt2 that senses CW damage and regulates the CW integrity pathway (Gandía et al., 2019), establishing a connection between CW, CW stress and the mechanism of AFPs.

In this report, we decided to expand the number of available null mutants of Chs in *P. digitatum* by characterizing mutants of ChsII and ChsV, which are relevant according to our previous gene expression studies, and to explore the role of chitin synthases in the mode of action of AFPs. Herein, we present data on *chs* gene expression in response to the treatment of *P. digitatum* with the self-AfpB. While the deletion of *chsII* did not seem to have major effects on the biology of the fungus beyond the production and morphology of conidia, the elimination of *chsV* generated mutant strains with severe alterations in growth and morphology, chitin content, reduction of conidia production and altered virulence. Interestingly, the two MMD-Chs mutants (Δ*chsV* and Δ*chsVII*) had differential phenotypes and showed altered sensitivity to both antifungal proteins, AfpA and AfpB.

## Materials and Methods

### Fungal Strains, Media and Culture Conditions

The *P. digitatum* parental strains used were the wild type CECT 20796 (Marcet-Houben et al., 2012) and the transformant PDMG314 (Δ*ku70:nat1*) (Gandía et al., 2016). All *P. digitatum* strains were routinely cultured on potato dextrose agar (PDA, Difco, Detroit, MI, United States) plates for 7–10 days at 24°C. Spores were collected, filtered, quantified with a hemocytometer, and adjusted to the appropriate concentration. Growth of strains and conidia production were evaluated by depositing 5 μL of a conidial suspension (5 × 10^4^ conidia/mL) on PDA plates, and the growth diameter was measured daily. Conidia production was determined as previously described (Gandía et al., 2014). Statistical analyses were conducted using the statistical SPSS 22.0 package (SPSS Inc., Chicago, IL, United States) to calculate the ANOVA test (*p* < 0.05). The *Agrobacterium tumefaciens* AGL-1 was used for fungal transformation (Gandía et al., 2014).

### Construction of Deletion Vectors and Fungal Transformation

To carry out the deletion of *chsII* and *chsV*, the double selection strategy (positive selection in hygromycin and negative in 5-fluoro-2’-deoxyuridine, F2dU) previously used in *P. digitatum* was performed (Gandía et al., 2014, 2016; Harries et al., 2015; Garrigues et al., 2016). Gene deletion constructs were obtained with the primers detailed in Supplementary Table S1. All PCR procedures were carried out with AccuPrime Taq DNA Polymerase, high fidelity (Invitrogen, Eugene, OR, United States), and the resulting DNA constructs were verified by DNA sequencing. Briefly, the hygromycin resistant cassette (*hph*) used as a positive selection marker, was amplified from pBHt2 (Khang et al., 2006) through PCR using the primers OJM197/OJM198. In the case of *chsII*, 1089 bp 5′ and 999 bp 3′ flanking regions were amplified from *P. digitatum* CECT 20796 genomic DNA with the primer pairs OJM408/OJM409 and OJM410/OJM411, respectively (Supplementary Figure S1). In the case of *chsV*, 1005 bp 5′ and 1026 bp 3′ flanking regions were amplified with the primer pairs OJM414/OJM415 and OJM416/OJM417, respectively (Supplementary Figure S2). Constructs obtained by Fusion PCR (Szewczyk et al., 2006) were cloned into pGKO2, and the obtained vectors pGKO2_Δ*chsII* and pGKO2_Δ*chsV* were transformed into *A. tumefaciens* AGL-1 by electroporation. CECT 20796 and PDMG314 (Δ*ku70*) strains were transformed by *A. tumefaciens*-mediated transformation (ATMT) as previously described (Khang et al., 2006) with minor modifications (Harries et al., 2015). Monosporic transformants were obtained through two rounds of selection on hygromycin-containing PDA plates and confirmed by PCR amplification of genomic DNA (Khang et al., 2006; Harries et al., 2015). The primers used were located at different positions around the target locus to discriminate and confirm the transformation events (Supplementary Table S1 and Supplementary Figures S1, S2). Ectopic strains, in which the transformed DNA was randomly inserted in a region different from the targeted locus, were recovered from each transformation experiment. These ectopic strains (PDMG2010 and PDMG612) were used in our experiments as additional controls.

### Phenotypic Characterization and Sensitivity Assays to Chemicals and Temperature

We tested the sensitivity of fungal strains to different compounds in 24-well cell culture plates (1 mL PDA per well). PDA medium was supplemented with 1.2 M sorbitol, 0.5 M NaCl, 150 μg/mL calcofluor white (CFW) (Fluorescent Brightener 28, Sigma-Aldrich, F3543), 150 μg/mL sodium dodecyl sulphate (SDS) (Sigma-Aldrich, L4509), 2 mM H_2_O_2_ and nikkomycin Z (NZ) (16, 64 and 128 μg/mL) (Sigma-Aldrich, N8028). Five μL of serial tenfold dilution of conidia (10^6^ to 10^3^ conidia/mL) were applied into each well. Plates were incubated at 24°C for 3–5 days. To test sensitivity to different temperatures, the different strains were grown on standard PDA plates at 24, 26, and 28°C for 7 days.

### Chitin Content

The determination of chitin content of fungal mycelia was based on methods described previously (Din et al., 1996; Gandía et al., 2014). Briefly, PDB cultures were inoculated with 2 × 10^5^ conidia/mL of *P. digitatum* strains and grown at 24°C for 5 days. Digestion was carried out with 0.2 U of chitinase suspension (Sigma-Aldrich, C6137) and 2 U of Zymolyase 20T (Euromedex, UZ1000). Data are the mean of four replicates and significance was determined by the statistical SPSS 22.0 package to calculate the ANOVA test (*p* < 0.05). The experiment was repeated twice with similar results.

### Microscopy Visualization

Each fungal strain was prepared, stained with CFW and microscopically visualized with a Nikon E90i fluorescence microscope as previously described (Garrigues et al., 2016). Bright field (BF) and fluorescence images were captured by the NIS-Elements BR v2.3 software (Nikon) and processed using FIJI software (Schindelin et al., 2012). To obtain the differential interference contrast (DIC) images of conidia, 100× objective was used and the images were captured, processed and analyzed by the NIS-Elements BR v2.3 software (Nikon). The Feret diameter (F, defined as the measure of an object size along a specified direction, or the distance between the two parallel planes restricting the object perpendicular to that direction) and the circularity (perimeter of the object) were measured to determine conidia shape. Statistical analyses were conducted using the statistical SPSS 22.0 package to calculate the ANOVA test (*p* < 0.05).

### Antifungal Assays

Growth inhibition assays were performed in 96-well microtiter plates (Nunc, Roskilde, Denmark) in a total volume of 100 μL as described previously (Garrigues et al., 2017) with modifications. Fifty μL of 2x conidia solution (5 × 10^4^ conidia/mL) in 1/10 diluted PDB medium containing 0.02% (w/v) chloramphenicol was mixed in each well with 50 μL of 2x concentrated protein solution (AfpA from *P. expansum* or AfpB from *P. digitatum*) from serial twofold dilutions (from 0.25 to 8 μg/mL for AfpA, or from 0.5 to 16 μg/mL for AfpB). All samples were prepared in triplicate and the mean and standard deviation (SD) were calculated. Plates were statically incubated for 4 days at 25°C, and growth was determined daily by measuring the optical density (OD) at 600 nm (OD600) using a FLUOstar Omega plate spectrophotometer (BMG labtech, Orlenberg, Germany). Dose-response curves were generated from measurements after 48 h. These experiments were repeated at least twice. The Minimum Inhibitory Concentration (MIC) is the peptide concentration that completely inhibited growth after 96 h of incubation, in all the experiments performed.

### Fruit Infection Assays

Fruit infection assays with different *P. digitatum* strains on mature freshly harvested orange fruits (*Citrus sinensis* L. Osbeck cv Navelina) were conducted as previously described (González-Candelas et al., 2010). Three replicates of five fruits were inoculated with 5 μL of different conidial suspensions (10^4^ and 10^6^ conidia/mL) at four wounds around the equator. Each inoculated wound was scored for green mold infection symptoms and data presented are the mean value ± SD of the percentage of infected wounds per replica at different days post-inoculation (dpi).

## Results

### Deletion of *chsII* and *chsV* in *P. digitatum*

We previously described and characterized seven chitin synthase genes in the *P. digitatum* genome (Gandía et al., 2012, 2014). A recent re-evaluation showed that *P. digitatum* also has a duplication of *chsIII* that would code for a second class III enzyme (ChsIIIb, PDIG_76160), as occurs with other fungi (Mellado et al., 1996; Choquer et al., 2004). In a previous work, we disrupted the MMD-containing class VII *chsVII* gene to obtain knockout mutants and demonstrate its involvement in CW integrity and virulence (Gandía et al., 2014). In the present study, and following the same approach, we obtained deletion mutants of another two *chs* genes to identify their functional role. First, we selected *chsV* that codes for the other MMD-Chs because of its increased expression levels during fungal infection. Additionally, we chose *chsII* because it is one of the most affected genes by the disruption of *chsVII* and due to its possible role in conidiogenesis, as it had been postulated for other class II c*hs* genes (Gandía et al., 2012; Sheng et al., 2013).

**FIGURE 1:**
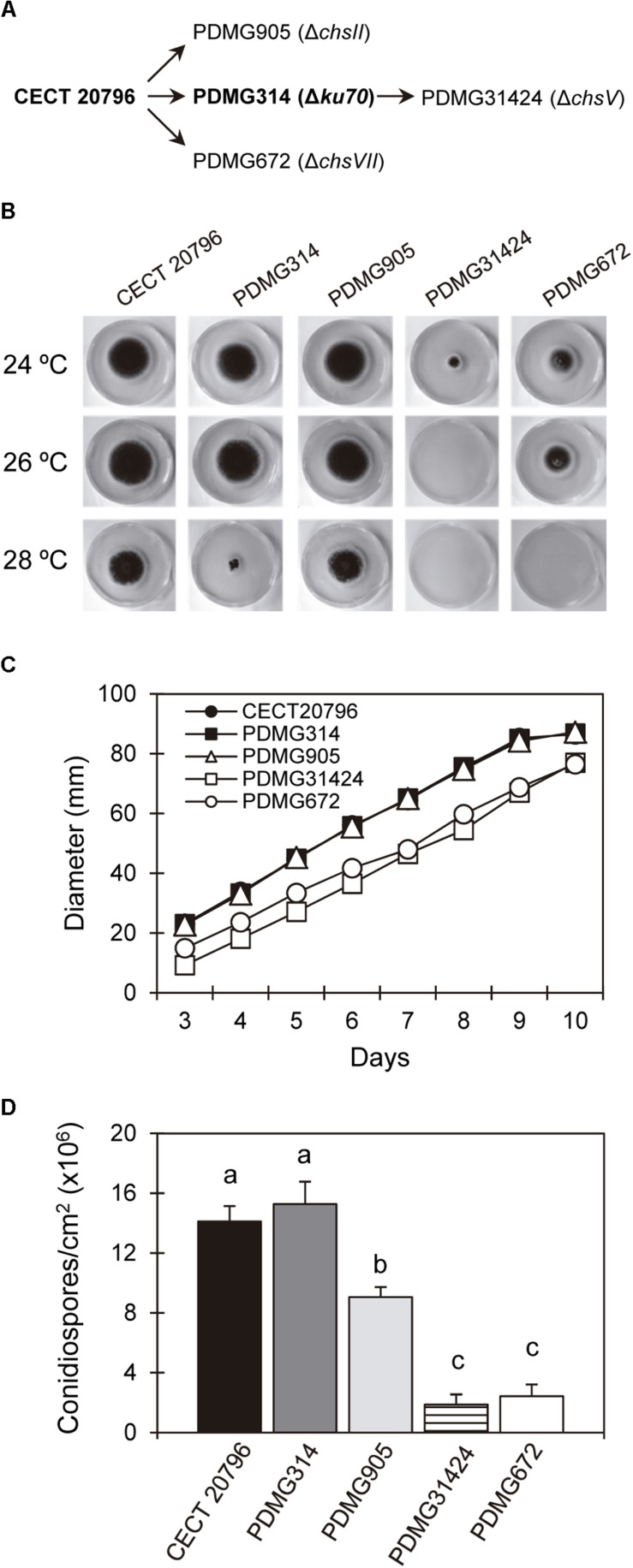
Growth and conidia production of different *P. digitatum* strains. **(A)** Schematic overview of the origin of different mutants, indicating the parental strains from which they come. **(B)** Radial growth at 7 days on PDA plates of the parental strains CECT 20796 and PDMG314 and deletion mutants Δ*chsII* (PDMG905); Δ*chsV* (PDMG31424) and Δ*chsVII* (PDMG672) at 24, 26, and 28°C. Note the absence of growth in PDMG31424 at 26 and 28°C and no growth of PDMG672 only at 28°C. **(C)** Colony diameter of different strains from 3 to 10 days of growth at 24°C in PDA. **(D)** Conidia production per surface area of different strains after 5 days of growth at 24°C on PDA plates. Data are mean values ± standard deviation (SD) of three replicate samples. Letters show significant differences among the strains within each day (ANOVA test, *p* < 0.05).

To obtain deletion mutants, a fragment of each gene was replaced by the *hph* cassette used as positive selection marker (Supplementary Figures S1A, S2A). Two different strains were used as parental: the wild type CECT 20796, and PDMG314 (Δ*ku70*) obtained to increase the homologous recombination (HR) frequency in *P. digitatum* by the inactivation of the Ku70/80 heterodimeric complex involved in the non-homologous end joining (NHEJ) DNA repair pathway (Gandía et al., 2016) (Figure 1A). The mutants Δ*chsII* and Δ*chsV* were mentioned in our previous publication to illustrate that a higher number of deletion strains were obtained when using PDMG314 as parental strain than with CECT 20796, demonstrating the increase of HR frequency (Gandía et al., 2016). Deletion mutants of *chsII* were easily obtained from both parental strains, and six of them (two obtained from the wild type, identified as PDMG905 and PDMG908 and four obtained from the PDMG314 strain, numbered between PDMG917 and PDMG926) were confirmed by a complete set of PCR amplifications with specific primers (Supplementary Table S1 and Supplementary Figure S1B). In the case of *chsV*, only two deletion transformants were obtained with PDMG314 as background and none with CECT 20796. The two deletion strains (PDMG31424 and PDMG31441) were also confirmed by PCR amplifications (Supplementary Figure S2B).

In the assays described below, we used independent mutants of each gene and found similar behavior in all of them, even among the *chsII* mutants obtained from two different backgrounds (data not shown). For simplicity, only one representative deletion strain of each gene is shown in most of the figures below: PDMG905 corresponding to Δ*chsII*, and PDMG31424 to Δ*chsV*. The functional complementation of these strains was not achieved: (i) in the Δ*chsII* mutant because it did not present major phenotypic differences with the wild type strain, (ii) in the Δ*chsV*, because –similar to the previous Δ*chsVII* (Gandía et al., 2014)– the CW and conidia production are so affected that impeded our attempts to obtain complemented strains.

### Phenotypic Characterization of Mutants

Deletion of many *chs* genes had, as a result, alterations in CW, growth, and sensitivity to temperature or in response to different stresses in different species as *Yarrowia lipolytica*, *P. digitatum* or *A. fumigatus* (Sheng et al., 2013; Gandía et al., 2014; Muszkieta et al., 2014). The growth and conidia production of deletion strains PDMG905 (Δ*chsII*) and PDMG31424 (Δ*chsV*) was compared with their respective parental strains CECT 20796 and PDMG314, and with the previously analyzed PDMG672 (Δ*chsVII*) as well (Figure 1). PDMG905 did not present differences in growth compared to the wild type parental (Figures 1B,C). Only PDMG31424 (Δ*chsV*) showed a strong reduction of growth at 24°C compared to the parental PDMG314, even more reduced than Δ*chsVII* strain (PDMG672).

*Penicillium digitatum* is very sensitive to temperature and cannot grow at 30°C or above. Figure 1B shows the sensitivity of the strains at the increasing temperatures of 24 (the usual in our laboratory), 26 and 28°C. When all strains where incubated at 28°C, a slight reduction of growth was similarly detected in the parental CECT 20796 and PDMG905 (Δ*chsII*). PDMG314 was clearly affected at 28°C, while PDMG672 and PDMG31424 were unable to growth at this temperature. At 26°C PDMG31424 did not grow either, contrarily to all the four other strains whose growth at 26°C was similar to that at 24°C (Figure 1B). We concluded that the deletion of *chsV* affected the temperature sensitivity of *P. digitatum* more than deletion of *chsVII*.

We next measured the production of conidia in the different strains (Figure 1D). Results revealed a significant but moderate reduction in conidia production of PDMG905 (Δ*chsII*), supporting the possible involvement of this gene in conidiogenesis. The mutants in the two MMD-Chs showed a strong and similar reduction of conidia production when the number of conidia was normalized to the surface of the colony (Figure 1D) (Gandía et al., 2014).

### Sensitivity of Mutants to Chemicals

**FIGURE 2:**
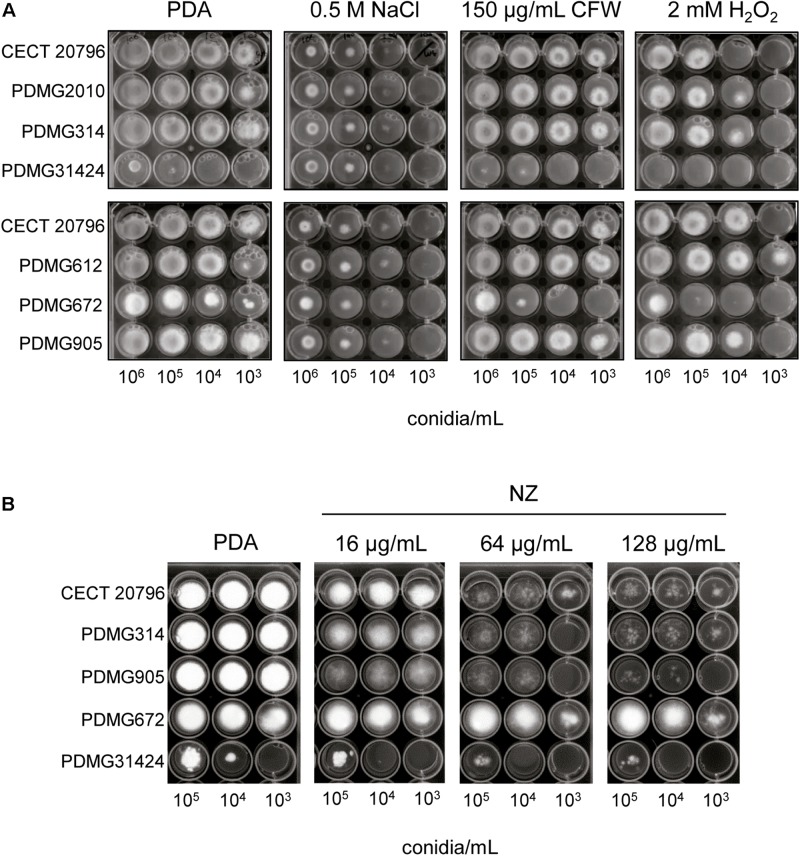
Sensitivity of *P. digitatum* strains to different compounds. **(A)** Comparison of different strains growth on PDA plates and PDA supplemented with the indicated compounds. Serial 1/10 dilutions of conidia of each strain were applied as indicated. **(B)** Growth of strains in presence of different concentrations of chitin synthase inhibitor nikkomycin Z (NZ) at 5 days of incubation at 24°C. Serial dilutions of conidia of each strain were applied as indicated. Different strains were: parental strains CECT 20796 and PDMG314; deletion strains: Δ*chsII* (PDMG905), Δ*chsV* (PDMG31424), and Δ*chsVII* (PDMG672) and two different ectopic strains PDMG2010 (*chsV*) and PDMG612 (*chsVII*).

To evaluate the sensitivity to chemicals of our mutants, we conducted growth assays in presence of different compounds. The osmotic stabilizer sorbitol recovered the growth of the deletion strains in *chsV* and *chsVII* (Supplementary Figure S3), as described previously in Δ*chsVII* mutants (Gandía et al., 2014). However, during osmotic stress caused by sodium salt (NaCl) only deletion mutant in Δ*chsV* was able to grow better than in PDA medium (Figure 2A).

The addition to PDA medium of compounds like CFW or SDS, which affect the CW and the cell membranes, respectively, produced a growth reduction in both Δ*chsV* and Δ*chsVII* mutants (Figure 2A and Supplementary Figure S3), indicative of CW damage. Similar test performed under oxidative stress (2 mM of H_2_O_2_) showed a higher sensitivity and a growth reduction of PDMG31424 (Δ*chsV*) (Figure 2A), similar to the results previously described for PDMG672 (Δ*chsVII*) (Gandía et al., 2014). In all these experiments, the strain PDMG905 (Δ*chsII*) did not differ in its growth profile in comparison with parentals CECT 20796 or PDMG314 (Figure 2A).

Treatments with the chitin synthase inhibitor NZ were conducted to determine changes in the sensitivity of the different strains. As shown in Figure 2B, the growth of the different strains was reduced in presence of distinct concentrations of NZ. Transformants Δ*chsII* and Δ*chsV* showed slightly higher sensitivity to NZ than that of their parental strains, whereas transformant PDMG672 (Δ*chsVII*) revealed increased resistance at all NZ concentrations tested.

### Morphology and Chitin Content of *chs* Mutants

**FIGURE 3:**
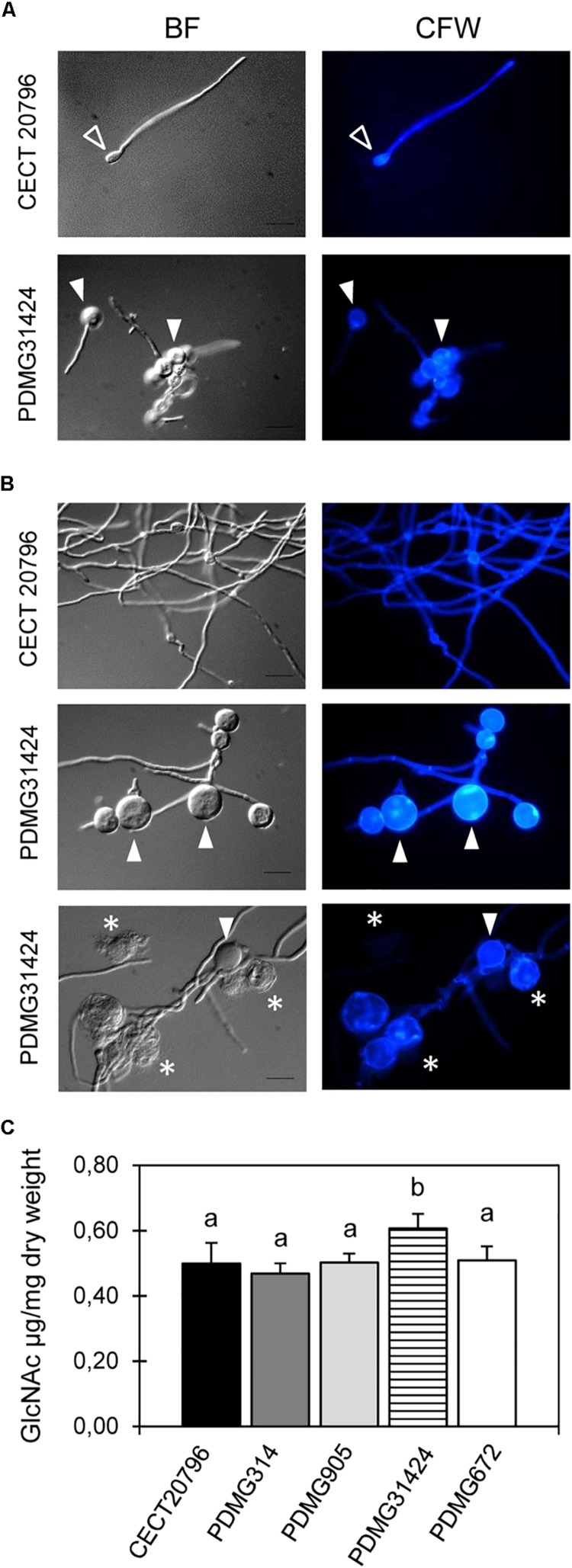
Morphological changes observed by microscopy and chitin content in *P. digitatum* strains. Representative images (BF, left and CFW stain, right) of fluorescence microscopy of the parental CECT 20796 and PDMG31424_Δ*chsV* strains at different times of incubation at 24°C. **(A)** 18 h, **(B)** 24 and 48 h. White arrowheads indicate balloon-like structures in mycelium of deletion strains intensively CFW stained and asterisks show shorter interseptal distances. **(C)** Chitin content assay of different strains determined as *N*-acetyl-D-glucosamine (GlcNAc) released per dry weight of tissue. Data are expressed as means ± SD of two replicates. Different letters indicate statistically significant differences among the strains as determined by ANOVA test *p* < 0.05. Scale black bars in BF images are 10 μm.

Changes in mycelial morphology of deletion mutants were analyzed by bright field and fluorescence microscopy under CFW staining that binds chitin. Mycelium of Δ*chsII* mutant strains (PDMG905) did not show morphological differences when it was compared with the two parental strains CECT 20796 or PDMG314 (data not shown). The mycelium of PDMG31424 (Δ*chsV*) exhibited strong microscopic alterations (Figures 3A,B). Germlings after 18 h of incubation presented shorter or aborted germ tubes and enlarged conidia (white arrowheads in Figure 3A); these conidia in the mutant were clearly larger than the usual enlargement that occurs in the wild type upon germination (open arrowheads in Figure 3A). At later times, balloon-like structures appeared and increased in size and number over time (arrowheads in Figure 3B), and were larger than similar structures observed previously in the Δ*chsVII* mutant (PDMG672) (Gandía et al., 2014). In addition, these balloon-like structures exhibited intense CFW staining and occasionally broke down and released the intracellular content (asterisks in Figure 3B). These structures and the increase in CFW staining are indicative of alterations in the CW and chitin deposition. The chitin content of the *chs* mutant strains was determined and PDMG31424 (Δ*chsV*) showed a statistically significant higher chitin content than in its parental or in the other strains (Figure 3C).

**FIGURE 4:**
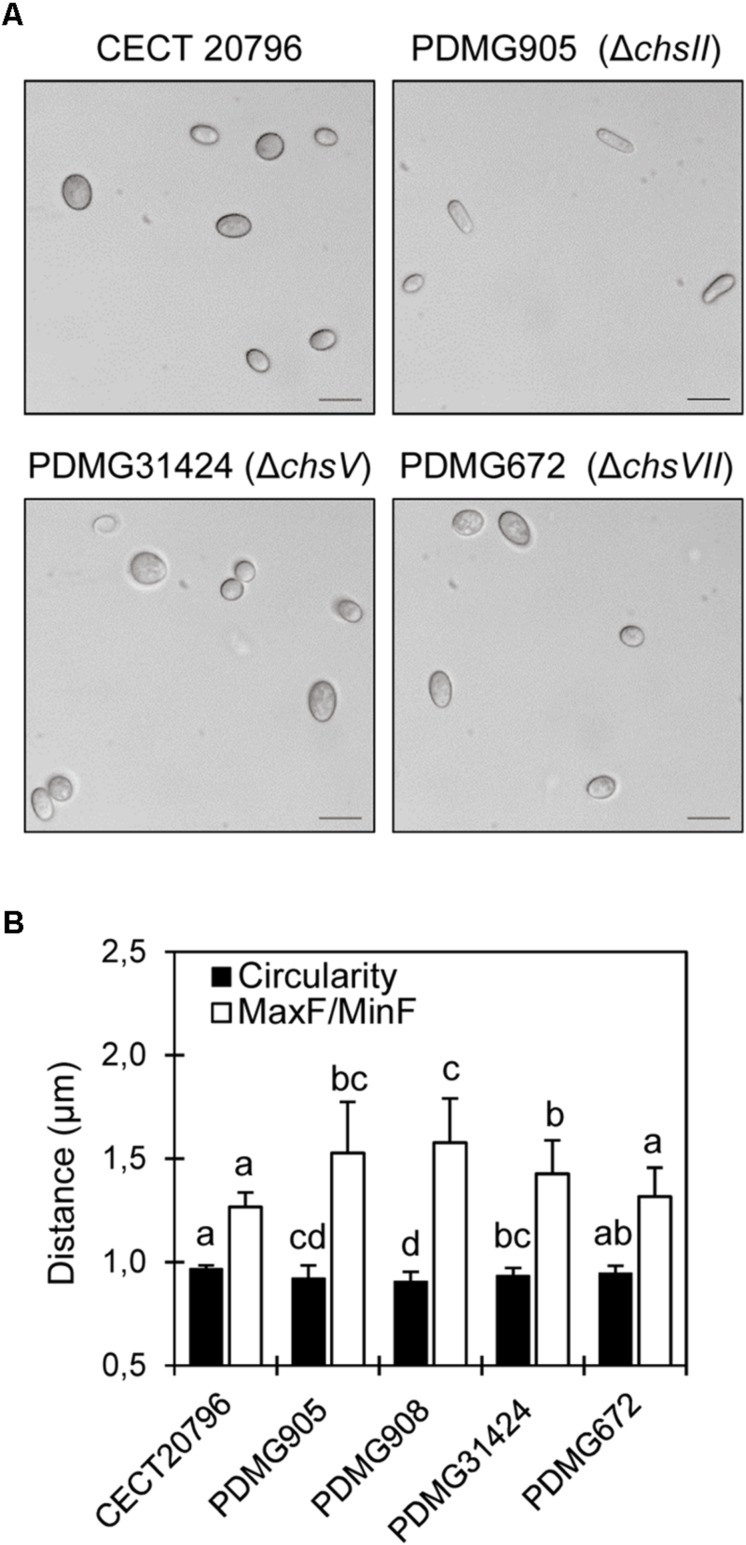
Phenotypic morphology and dimensions of spores of different *P. digitatum* strains. **(A)** Differential interference contrast (DIC) images obtained with 100x objective, of conidia of different strains. Scale black bars are 10 μm. **(B)** Analyses of two different parameters of conidia (F and circularity) in different strains. Both PDMG905 and PDMG908 are Δ*chsII* deletion strains. Different letters indicate statistically significant differences among the strains determined by ANOVA test *p* < 0.05.

The conidia of the different strains were observed at 100× objective and, at first sight, Δ*chsII* conidia were clearly elongated compared with the parental and other mutants (Figure 4A). In an attempt to quantify any differences in the morphology of conidia, two shape parameters were analyzed in distinct mutants. The Feret diameter (F) and the circularity were measured to verify the difference among conidia shape and size in Δ*chsII* and other deletion mutants. Both parameters confirmed differences in two independent Δ*chsII* mutants (PDMG905 and PDMG908), more pronounced than in the Δ*chsV* mutant (PDMG31424) as compared to the parental (Figure 4B). PDMG314 and CECT 20796 parental strains did not present any differences between them (data not shown).

### Effect of Antifungal Proteins on *chs* Gene Expression and Deletion Mutants

Previously, chitin synthesis and chitin synthase genes have been linked to the sensitivity of filamentous fungi to AFPs (Hagen et al., 2007; Martín-Urdiroz et al., 2009). We have recently managed to produce and purify in high amounts two highly active antifungal proteins (AfpA and AfpB) (Garrigues et al., 2017, 2018), offering a great opportunity to evaluate the role of chitin synthase genes in the mode of action of these proteins. Side-by-side experiments have shown that these two AFPs are more active *in vitro* against *P. digitatum* than the previous AFP from *A. giganteus* and PAF from *P. chrysogenum*, and that both AfpA and AfpB show protective effect against the infection of fungal plant pathogens (Garrigues et al., 2017, 2018; Shi et al., 2019).

We have determined global gene expression changes by RNA-seq after treatment of *P. digitatum* with the self-AfpB (Bolós et al., unpublished). Table 1 shows the fold changes in gene expression corresponding to the *chs* genes, data extracted from the global gene expression experiment. In these data sets, treatments A and B correspond, respectively, to 1 and 3 h treatments of previously 24 h-grown *P. digitatum* mycelium with sub-inhibitory concentrations of AfpB; while treatment C corresponds to *P. digitatum* grown for 24 h in the continuous presence of sub-inhibitory concentrations of AfpB. An overall reduction of *chs* gene expression is observed that only reached statistical significance in treatment C and in the case of *chsI*, *chsII*, and *chsIIIb*.

**TABLE 1:** Gene expression changes of the genes coding for chitin synthases after AfpB treatments determined by RNA-seq.

		**Treatment A^a^**			**Treatment B^a^**			**Treatment C^a^**		
				
**Gene**	**ID**	**log_2_FC^b^**	**log_2_CPM^b^**	**FDR^b^**	**log_2_FC**	**log_2_CPM**	**FDR**	**log_2_FC**	**log_2_CPM**	**FDR**
*chsI*	PDIG_90720	0.63	8.39	7.1E-01	−0.30	7.91	8.1E-01	**−2.66^∗^**	**7.13**	**6.4E-08**
*chsII*	PDIG_23050	−0.13	6.54	9.8E-01	0.09	6.67	9.6E-01	**−0.79^∗^**	**6.13**	**2.2E-02**
*chsIIIa*	PDIG_22170	0.06	9.06	1.0E + 00	0.05	9.08	1.0E+00	−0.54	8.66	2.2E-01
*chsIIIb*	PDIG_76160	0.44	7.82	7.7E-01	0.00	7.60	1.0E+00	**−2.31^∗^**	**6.72**	**4.8E-11**
*chsIV*	PDIG_89090	−0.39	7.41	8.0E-01	−0.29	7.47	7.1E-01	−0.15	7.40	7.8E-01
*chsV*	PDIG_69930	−0.04	8.82	1.0E + 00	−0.56	8.61	3.8E-01	−0.68	8.42	1.3E-01
*chsVI*	PDIG_78580	−0.37	2.58	8.8E-01	−0.58	2.50	4.4E-01	−0.87	2.25	7.2E-02
*chsVII*	PDIG_69920	−0.23	8.88	9.5E-01	−0.56	8.76	4.3E-01	−0.74	8.54	1.1E-01

*^*a*^Treatments A and B: 24 h-grown *P. digitatum* mycelium treated with 1 μg/mL AfpB for 1 h (A) or 3 h (B). Treatment C: *P. digitatum* mycelium grown for 24 h in the presence of 0.03 μg/mL AfpB. ^*b*^FC, Fold-expression Change between each treatment (A, B, or C) and the control treatment with no AfpB protein added; CPM, sum of Counts Per Million of reads of each treatment plus the control treatment; FDR, False Discovery Ratio as determined by the EdgeR software. ^∗^Significant gene expression changes as determined by FDR < 0.05 are marked in bold.*

**FIGURE 5:**
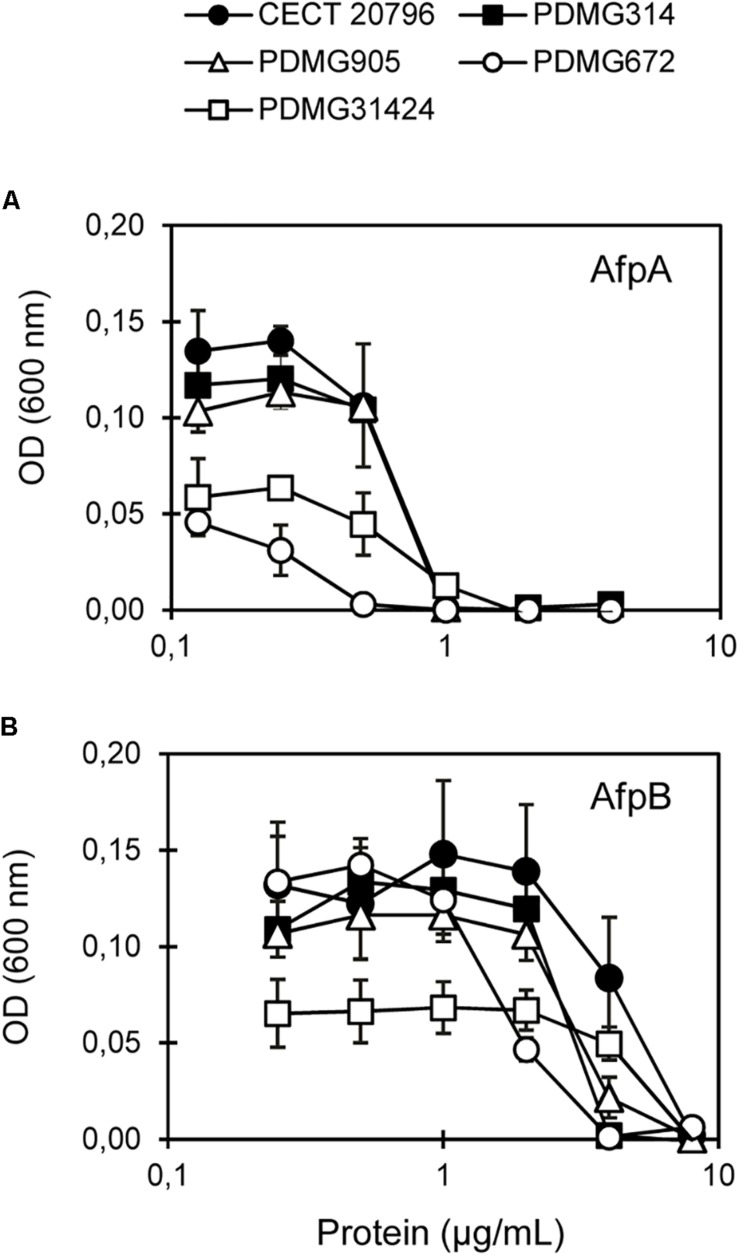
*In vitro* inhibitory activity of antifungal proteins against *chs* mutants. Dose-response curves of antifungal proteins against parental strains (CECT 20796 and PDMG314) and *chs* mutants (PDMG905_Δ*chsII*; PDMG672_Δ*chsVII* and PDMG31424_Δ*chsV*). **(A)** Response to AfpA. **(B)** Response to AfpB. Curves show mean ± SD after 48 h at 25°C. Strains showed: CECT 20796 black circles; PDMG314 black squares; PDMG905 white triangles; PDMG31424 white squares and PDMG672 white circles.

Next, we assayed the effect of the AfpB from *P. digitatum* and AfpA from *P*. *expansum* on the Chs mutants. Dose-response curves at 48 h of growth (Figure 5) and MIC values at the end of experiments (Table 2) showed differences among mutants. The Δ*chsII* mutant exhibits a behavior similar to that of the parental strain, in spite of the significant gene expression change of this gene in the presence of AfpB (Table 1). On the other hand, the MMD-chitin synthase mutant PDMG672 (Δ*chsVII*) showed increased sensitivity to both proteins in all the experiments carried out. At the end of the experiment (72 h), growth of PDMG672 was completely inhibited at concentrations of AfpB and AfpA twofold lower than the parental or other strains, and at lower concentrations of proteins or shorter incubation times the growth of this mutant was affected. This sensitivity was more accused in the case of the more active AfpA (establishing a MIC of 0.5 μg/mL) (Table 2). In the case of the other MMD-chitin synthase mutant PDMG31424 (Δ*chsV*) the data were more difficult to interpret, due to the poor growth of this mutant in liquid medium; see for instance its reduced growth at non-inhibitory 0.25 μg/mL AfpB in Figure 5B, in comparison with the other strains including its parental PDMG314. In any case, the mutant behaved as its parental strain in some experiments with the same MIC while in others showed slightly increased tolerance with a twofold increase of MIC. Nevertheless, in some experiments, this Δ*chsV* had increased OD values over its parental at intermediate protein concentrations and times (see for instance white and black squares in Figure 5B).

**TABLE 2:** Minimal inhibitory concentration (MIC) values (μg/mL) of two antifungal proteins AfpA and AfpB against different fungal strains tested.

	**AfpA (μg/mL)**		**AfpB (μg/mL)**	
		
	**Exp^a^. 1**	**Exp^a^. 2**	**Exp^a^. 1**	**Exp^a^. 2**
CECT 20796	2	1	4	8
PDMG905 (Δ*chsII*)	2	1	4	8
PDMG672 (Δ*chsVII*)	0.5	0.5	2	4
PDMG314 (Δ*ku70*)	2	1	8	4
PDMG31424 (Δ*ku70*; Δ*chsV*)	2	2	8	8

*^*a*^MIC values from two independent experiments are shown.*

### Comparison of Virulence of *chs* Mutants

**FIGURE 6:**
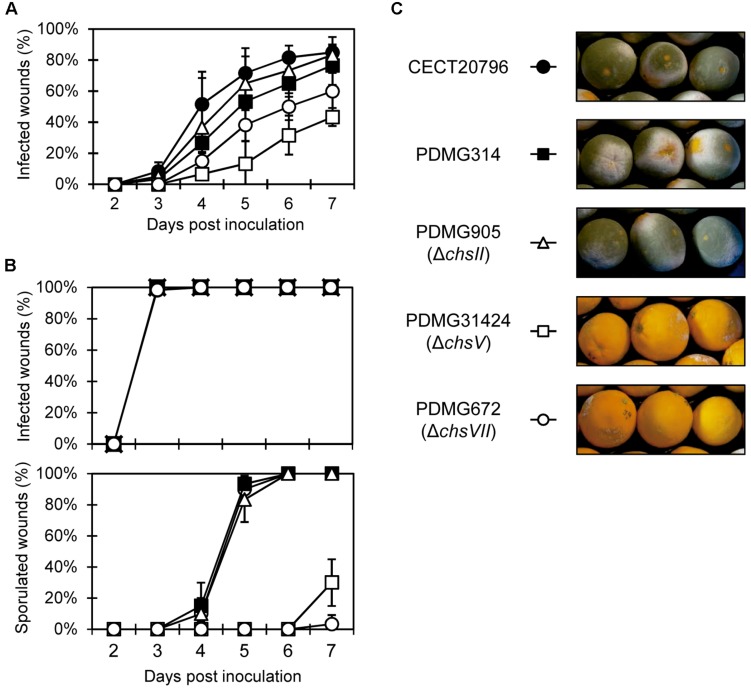
Virulence assays of different *P. digitatum* strains on orange fruits. **(A)** Incidence of infection caused by different strains at low inoculum dose (10^4^ conidia/mL). Data indicate percentage of infected wounds (mean value ± SD) at each day post-inoculation. **(B)** Incidence of infection caused by different strains, at high inoculum dose (10^6^ conidia/mL) (top panel) and percentage of sporulated wounds on fruits from the same experiment (bottom panel). **(C)** Representative images of fruits infected with different strains at 7 days post-inoculation from the experiment shown in **(B)**. Note the absence of mycelia in fruits infected with Δ*chsV* and Δ*chsVII* deletion strains.

Finally, we analyzed the virulence of the different *chs* mutants by conducting experimental inoculation assays on orange fruits with different inoculum doses (Figure 6). Low inoculum doses (10^4^ conidia/mL) confirmed that the deletion strain PDMG905 (Δ*chsII*) had the same incidence of infection as parental strains CECT 20796 and PDMG314. However, the deletion strains in MMD-chitin synthase genes *chsV* and *chsVII* had a low incidence of infection, lower in PDMG31424 than in PDMG672 (Figure 6A). At seven dpi, between 75 and 85% of wounds were infected in both parental strains and PDM905; however, PDMG672 (Δ*chsVII*) mutant reached 60% of infection while PDMG31424 (Δ*chsV*) was only able to infect 43% of wounds (Figure 6A). Both mutants showed reduced maceration area in citrus fruits (data not shown). Previous analyses had shown reduced virulence in the strain PDMG672, confirmed again in this work (Gandía et al., 2014). When higher inoculum doses were used (10^6^ conidia/mL) all strains developed symptoms quickly and reached 100% of infection incidence at 3 dpi (Figure 6B top panel). The maceration area on citrus fruits was the same in all strains analyzed under these inoculum conditions (data not shown), and the sporulation index of infected wounds was specifically reduced in Δ*chsV* and Δ*chsVII* mutants (Figures 6B bottom panel and Figure 6C). At the end of the experiments (7 dpi), citrus fruits infected by Δ*chsV* and Δ*chsVII* at 10^6^ conidia/mL were 100% infected and completely macerated although most of them did not develop green conidia, thus confirming that the Δ*chsV* mutants have a phenotype similar to that of the previously described Δ*chsVII* (Gandía et al., 2014) (Figures 6B,C).

## Discussion

The role of chitin synthase genes in fungal development and pathogenesis has been studied through gene deletions in a wide number of fungi, and the general conclusions are common in many fungal species, with just minor differences (Morcx et al., 2013; Gandía et al., 2014; Muszkieta et al., 2014; Liu et al., 2016; Zhang et al., 2016). However, the involvement of *chs* genes and chitin biosynthesis process in the sensitivity to AFPs from fungal origin remains controversial (Hagen et al., 2007; Martín-Urdiroz et al., 2009; Ouedraogo et al., 2011).

Our previous disruption of the *chsVII* gene of *P. digitatum* showed important effects on growth, conidia production or virulence and compensatory changes of expression in other *chs* genes, indicative of redundancy or cooperative role among different *chs* (Gandía et al., 2014), similar to other filamentous fungi (Lenardon et al., 2010; Sánchez-Vallet et al., 2014). In order to study the role of *chsII*, which is one of the most induced gene by *chsVII* disruption, and the role of the other MMD-*chs* gene (*chsV*), we obtained viable mutants in this study. Both of them belong to different classes and divisions, and results could indicate the different role of each Chs division in this fungus and could help to clarify the mechanism of action of antifungal proteins.

### The Mutation of *chsII* in *P. digitatum* Reduced the Number and Changed the Morphology of Conidia

*Penicillium digitatum* mutants in the *chsII* gene were similar to the parental strain in many aspects. There were no morphological changes in growth or sensitivity to temperature (Figure 1), sensitivity to different compounds (Figure 2), chitin content (Figure 3), sensitivity to antifungal proteins AfpA or AfpB (Figure 5) or virulence (Figure 6). The phenotype of class II Chs mutants is subtle, and did not show significant growth alterations in many fungi such as *Aspergillus nidulans* (Motoyama et al., 1997), *Aspergillus fumigatus* (Munro and Gow, 2001), or *Neurospora crassa* (Fajardo-Somera et al., 2015). Gonçalves et al. (2016) explained that the single mutants in class I or class II did not have changes in growth or morphology due to redundant roles of these Chs classes. In the division I overlapping and cooperative functions of Chs are known, since the generation of double or multiple mutants in Chs belonging to this division produces abnormal morphologies. Double mutants in *chsI/chsII* in *A. nidulans* presented a dramatic decrease in conidia production not observed in the single mutants, despite the normal content of chitin (Fujiwara et al., 2000; Ichinomiya et al., 2005). Quadruple mutant Δ*chsA*/*C*/*B*/*G* in *A. fumigatus* showed reduced conidiation, growth and Chs activity (Muszkieta et al., 2014). In other studies, conducted in *M. oryzae* or *A. nidulans*, a role of this gene in conidiogenesis has been described based on the low conidia production of single mutants (Culp et al., 2000; Fujiwara et al., 2000; Kong et al., 2012). In *P. digitatum* a possible role of *chsII* in conidiogenesis was established on the basis of its strong induction during sporulation on PDA plates (Gandía et al., 2012). The mutants reported here in *chsII* showed a 30-40% reduction in the number of conidia (Figure 1) and abnormalities in conidia morphology (Figure 4) that demonstrate a role in conidiogenesis despite normal chitin content (Figure 3). In *Wangiella dermatitidis*, class II Chs was established as responsible for normal reproductive growth and mutants in this gene had sometimes enlarged cells (Zheng et al., 2006). All of these results support the possible involvement of class II Chs in *P. digitatum* conidiogenesis.

### Disruption of MMD-Chitin Synthases Alters Morphology and Virulence of *P. digitatum*

A number of studies have described the important role of the MMD ChsV and ChsVII chitin synthases in the development and virulence of fungal plant pathogens (Kim et al., 2009; Treitschke et al., 2010; Larson et al., 2011; Kong et al., 2012; Fernandes et al., 2016). Previously, we obtained and characterized *P. digitatum* disruption strains in *chsVII* (Gandía et al., 2014). We did not obtain mutants in *chsV* in the first attempts using the wild type CECT 20796 as parental strain. We only succeeded after using a strain (PDMG314) with the non-homologous end-joining pathway (NHEJ) inactivated by deletion of *ku70* gene involved in this route (Gandía et al., 2016). The increased gene targeting efficiency in *P. digitatum* using this strain allowed the generation of disruption mutants in *chsV*, which showed phenotypic alterations similar but more accused than Δ*chsVII* mutants, in accordance with other class V mutants that showed strongest phenotypes (Roncero et al., 2016).

Both mutants (Δ*chsV* and Δ*chsVII*) had reduced growth and conidia production, increased sensitivity to CFW, H_2_O_2_ and temperature stress, and showed alterations in the chitin content and hyphal morphology (Figures 1–3). Moreover, the Δ*chsV* mutant shows extremely enlarged balloon-like cells that eventually break out and release the intracellular material (Figure 3B). The number and size of these structures are higher than those described for Δ*chsVII* (Gandía et al., 2014), and both are intensely stained with CFW and could not be restored by osmotic stabilizers. These structures are common in other class V/class VII mutants (Takeshita et al., 2006; Jiménez-Ortigosa et al., 2012; Muszkieta et al., 2014).

All these phenotypic effects are indicative of alterations in CW (Munro, 2013; Walker et al., 2015) and, consequently, growth was recovered with osmotic stabilization with sorbitol or NaCl (Figure 2 and Supplementary Figure S3). Similar defects were present in ChsV/ChsVII mutants obtained in other filamentous fungi such as *A. fumigatus* (Muszkieta et al., 2014), *A. nidulans* (Takeshita et al., 2006), *B. cinerea* (Morcx et al., 2013), *Fusarium oxysporum* (Martín-Urdíroz et al., 2008), or *M. oryzae* (Kong et al., 2012). Several studies have pointed out the role of MMD-Chs in the hyphal growth, being transported by vesicles along actin filaments to the apical hyphae wherein CW-forming foci are located and contributing to polarize CW synthesis (Schuster et al., 2012; Fajardo-Somera et al., 2015; Takeshita et al., 2015). The apical growth of Δ*chsV* is heavily affected in germlings of the mutants (Figure 3A).

*Penicillium digitatum* shows temperature sensitivity. It grows and germinates at 24–25°C as optimal temperature, but at temperatures above 25°C the levels of germination are reduced up to 40–60% and at 30°C it is unable to germinate or grow (Plaza et al., 2003). The disruption of *chsV* using Δ*ku70* as genetic background produced strains with extreme temperature sensitivity, part of which could be explained by the sensitivity of the parental strain from which it proceeds (Gandía et al., 2016). The class V Chs protein in *W. dermatitidis* is necessary for growth of this fungus at 37°C maintaining the integrity of hyphal tip (Liu et al., 2004; Abramczyk et al., 2009). Also, mutants of *A. fumigatus* in MMD-*chs* genes showed increased sensitivity to high temperature (Muszkieta et al., 2014).

It has been described that the perturbations of CW caused by mutations in *chs* genes trigger compensatory mechanisms such as the increase in chitin synthesis and content (Munro et al., 2007; Roncero et al., 2016). In *P. digitatum*, the mutant Δ*chsV* was again remarkable in the abnormal and high chitin content compared to wild type (Figure 3C). However, we did not find any correlation between the chitin content and sensitivity to the chitin biosynthesis inhibitor NZ. This antifungal inhibits chitin synthesis by preventing the conversion of UDP-*N*-acetylglucosamine into chitin. Treatment with this inhibitor revealed slight sensitivity in Δ*chsII* and Δ*chsV* (Figure 4). Both strains had different chitin levels, higher in Δ*chsV* (Figure 3). Only Δ*chsVII* registered increased resistance to NZ and its chitin content was similar to the wild type. This pattern of NZ sensitivity was not comparable to other fungi. For instance, *M. oryzae* mutants in class II (Chs2) and class V (Chs6) were more sensitive to NZ as in *P. digitatum*, but mutants in class VII (Chs5) were not different from wild type (Kong et al., 2012). In addition, none of the Chs mutants had significant changes in the chitin content (Kong et al., 2012). None of the MMD-Chs mutants in *A. fumigatus* showed differences in susceptibility to NZ either (Jiménez-Ortigosa et al., 2012).

Many studies have suggested the role of MMD-chitin synthases in pathogenicity or virulence (Fernandes et al., 2016). In our study, both MMD-mutants showed reduced virulence in citrus fruits (Figure 6) confirming this general behavior also in a fruit-specific pathogen. The incidence of infection was lower in Δ*chsV* mutant than in Δ*chsVII*, pointing again to the more extreme phenotype of the former. As in the case of Δ*chsVII* (Gandía et al., 2014), the mutant in class V showed delayed disease progression and was unable to develop mycelium in most of the infected wounds in spite of the massive colonization of the fruit tissue. These results confirmed the prominent role of these chitin synthases in virulence of *P. digitatum*, since are the most induced *chs* genes during infection of citrus fruits (Gandía et al., 2014). Altered virulence was demonstrated in other phytopathogenic fungi when MMD-Chs were eliminated (Weber et al., 2006; Treitschke et al., 2010; Larson et al., 2011; Cui et al., 2013), but, again, subtle differences were observed in fungi such as *M. oryzae* in which the class V was non-pathogenic but the class VII mutant behaved as the wild type (Kong et al., 2012). In *F. oxysporum*, the inability of mutants in MMD-*chs* to invade tomato plants was linked to their hypersensitivity to hydrogen peroxide, compound produced as part of the plant defense response (Madrid et al., 2003). On the contrary, *Ustilago maydis* mutants in class V lost the ability to infect, but were not affected in their sensitivity to H_2_O_2_, so the absence of virulence does not seem to be due exclusively to a lack of response to plant defense (Weber et al., 2006; Treitschke et al., 2010). Despite this, it seems that these MMD-chitin synthases could be involved in response to host defense mechanism (Fernandes et al., 2016). In our case, both MMD-mutants were more sensitive to H_2_O_2_ than parental strains. *P. digitatum* is able to neutralize the ROS response that citrus fruit generate upon infection (Macarisin et al., 2007; Costa et al., 2019). In our previous study, we speculated that the high sensitivity of Δ*chsVII* mutant to H_2_O_2_ was related to its decreased virulence (Gandía et al., 2014). The fact that another H_2_O_2_ sensitive mutant (Δ*chsV*) also has reduced virulence whereas Δ*chsII* with a sensitivity to H_2_O_2_ similar to the parental strain does not present changes in its virulence would further support this hypothesis.

### Differential Sensitivity of MMD-Chs Mutants to Antifungal Proteins and Peptides

Antimicrobial peptides and proteins (AMPs) are a promising alternative in the fight against fungal pathogens (Hegedüs and Marx, 2013). Some of these peptides and proteins may have modes of action different from available fungicides. The involvement of CW in the interaction of AMP/AFPs with fungi is well documented. Upon interaction with the fungal cell, the *A. giganteus* AFP localizes first at the outer CW rich in glycoproteins (Theis et al., 2005), from where it is actively internalized (Oberparleiter et al., 2003). Other AFPs also locate intracellularly (Sonderegger et al., 2017; Huber et al., 2018), and we have similar observations for the AfpB from *P. digitatum* (Marcos et al., unpublished).

The observation that the antifungal protein AFP from *A. giganteus* can be purified by chitin affinity chromatography (Liu et al., 2002) sustained the early hypothesis that chitin or chitin synthesis are important factors in the mode of action of AFPs or in the response mechanisms of fungi to these proteins (Hagen et al., 2007). However, some contradictory results were found in the studies published so far with this AFP (Hagen et al., 2007; Martín-Urdiroz et al., 2009; Ouedraogo et al., 2011), since the results reported in these works do not completely correlate with our own observations described here. Exposure to *A. giganteus* AFP inhibited the biosynthesis of chitin in several sensitive filamentous fungi (Hagen et al., 2007). Chitin staining with CFW decreased and was altered in the sensitive *A. nidulans* treated with PAF (Binder et al., 2010) or *Aspergillus flavus* with PgAFP (Delgado et al., 2015). Down regulation of *chs* genes observed upon treatment of the sensitive *P. digitatum* with its self-AfpB (Table 1) is consistent with the inhibition of Chs activity observed in sensitive filamentous fungi treated with AFP (Hagen et al., 2007).

In addition, mutants in Δ*chsIII* and Δ*chsV* from *F. oxysporum* or *A. oryzae* were reported to be highly resistant to AFP, with a MIC from 1 to 400 μg/mL in the case of *F. oxysporum* Δ*chsV*. Interestingly, AFP did not inhibit chitin biosynthesis in the Δ*chsV* resistant strain but rather induced chitin synthase activity (Hagen et al., 2007). In contrast, a different study showed higher sensitivity to this protein in the same *F. oxysporum* Δ*chsV* strain (Martín-Urdiroz et al., 2009).

Ours is the first study that analyzes and compares the sensitivity to two AFPs in mutants from both MMD-containing Chs and provides data on ChsVII. Moreover, AfpA and AfpB are highly active AFPs, and AfpB was identified in the *P. digitatum* strain that is used for mutant generation. This context offers a great opportunity to refine the role of chitin biosynthesis in the action of AFPs. When our *P. digitatum chs* mutants were exposed to either AfpA or AfpB, only one of them (Δ*chsVII*) presented a subtle but clear differential response, with sensitivity twofold higher than CECT 20796 or PDMG314 parental strains (Figure 5). In this study, the Δ*chsV* presented an even higher chitin content than Δ*chsVII*. It was difficult to conclude any differential behavior of this mutant in terms of sensitivity to the proteins due to its growth penalty; if any, it is of increased tolerance that, however, did not reach the extreme resistance of *F. oxysporum* Δ*chsV* (Hagen et al., 2007).

Echinocandins are antifungals used in clinic. They are cyclic lipo-hexapeptides that are produced by ascomycetous fungi through non-ribosomal peptide synthases that target the CW by inhibiting β-glucan synthesis (Emri et al., 2013). The mutation of MMD-Chs (CsmA and CsmB) in *A. fumigatus* produced extreme sensitivity to several echinochandins including caspofungin (Jiménez-Ortigosa et al., 2012). We have also developed and characterized the small synthetic hexapeptide PAF26 (Muñoz et al., 2013). As occurs with AFPs, PAF26 firstly interacts with CW and is later internalized by the fungal cell to produce the cytotoxicity and antifungal effect intracellularly (Marcos and Gandía, 2009; Muñoz et al., 2013). Thus, the CW is critical for the interaction and activity of AFPs and other antifungal peptides such as PAF26. Defects in CW and protein mannosylation in the protein *O*-mannosyltransferase mutant Δ*pmt2* increase the tolerance of *P. digitatum* to PAF26 (Harries et al., 2015). On the contrary, the Δ*chsVII* mutant did not show differential sensitivity to PAF26 compared to the parental strain (Gandía et al., 2014). This is in contrast with the increased sensitivity of this mutant to the AFPs tested in this study.

The comparison between Δ*chsV* and Δ*chsVII* of growth phenotype, the morphological alterations in the hyphae, temperature sensitivity or decreased tolerance to CW interfering compounds suggest a more severe CW alteration in Δ*chsV*, which does not correlate with the relative sensitivity to either AfpA or AfpB, indicating that CW may not be an important (or unique) determinant for sensitivity to AFPs.

The mode of action and effects of the *A. giganteus* AFP were also analyzed in the model unicellular fungus *S. cerevisiae*, which is tolerant to the protein, with no growth inhibition up to 400 μg/mL AFP (Ouedraogo et al., 2011). The authors showed that AFP activates among others the CW integrity (CWI) pathway and induces chitin content and the expression of the three *chs* genes found in yeast. We have shown induction of the CWI pathway by AfpB (Gandía et al., 2019). Treatment with AfpB induces phosphorylation of Slt2 and Hog1 MAPK, revealing the involvement of CWI and high osmolarity glycerol (HOG) pathways in its mechanism of action, but not in defensive roles, since mutants in these routes were more tolerant to the action of the protein (Gandía et al., 2019). The increased gene expression in yeast exposed to AFP contrasts with the overall repression we observed with *P. digitatum*. The data reported here reinforces the conclusion that an increased chitin response is found in AFP-resistant fungi (*S. cerevisiae* or *F. oxysporum* Δ*chsV*) while AFPs reduce chitin biosynthesis in sensitive filamentous fungi (Ouedraogo et al., 2011). However, the exact role of *chs* gene mutation is still a matter of debate and needs to be further analyzed in more depth since different studies including this one have led to different conclusions.

Previous studies have pointed the role of MMD-Chs at the growth region of the hyphal apical tip (Steinberg, 2011; Schuster et al., 2016; Geoghegan et al., 2017). Cooperation of both MMD-Chs is needed for the final step of exocytosis that would form the new glucan and chitin chains in the growing hyphae (Geoghegan et al., 2017). Future studies with fluorescence tagged Afps would provide evidence of their location in the hyphae of parental and mutant strains and whether this location matches that described for MMD-Chs. These assays would help us to understand the mechanism of action of these antifungal proteins and the chitin biosynthesis response in sensitive or resistant strains to Afps.

Our results show that *chs* genes belonging to different divisions of Chs are responsible for different aspects in *P. digitatum* biology. Deletion of ChsII did not produce significant changes in the morphology or virulence of the fungus but affected its conidia formation process. Mutants in MMD-Chs from division II compromised growth, morphology, and virulence of the fungus, and influenced the sensitivity to antifungal proteins. Further studies about changes in *P. digitatum* in response to AFPs exposure will be addressed in the near future.

## Data Availability Statement

All datasets generated for this study are included in the manuscript/Supplementary Files.

## Author Contributions

MG, PM, and JM conceived and designed the study. MG obtained the different mutants. MG and SG phenotypically characterized the mutants. BB and JM performed the RNA-seq experiments and analyses. SG and PM obtained and purified the proteins. MG and JM prepared the first draft of the manuscript. PM and JM provided the funding. All authors contributed to manuscript revision, read and approved the submitted version.

## Conflict of Interest

The authors declare that the research was conducted in the absence of any commercial or financial relationships that could be construed as a potential conflict of interest.
