# Supplementary material for: The Myosin Motor Domain-Containing Chitin Synthases Are Involved in Cell Wall Integrity and Sensitivity to Antifungal Proteins in Penicillium digitatum
Source: Front Microbiol. 2019 Oct 18;10:2400. doi: 10.3389/fmicb.2019.02400 (PMC6813208; doi:10.3389/fmicb.2019.02400)
Supplement: Supplementary file 1 [file Data_Sheet_1.pdf]

Supplementary Material

The myosin motor domain-containing chitin synthases are involved in cell wall integrity and sensitivity to antifungal proteins in *Penicillium digitatum*.

Mónica Gandía[1,*], Sandra Garrigues[1±], Begoña Bolós[1], Paloma Manzanares[1] and Jose F. Marcos[1]

[1]Department of Biotechnology, Instituto de Agroquímica y Tecnología de Alimentos (IATA), Consejo Superior de Investigaciones Científicas (CSIC), Paterna, Valencia, Spain

*** Correspondence:** Mónica Gandía: mgandia@iata.csic.es

1. Supplementary Figures and Tables

1.1 Supplementary Figures

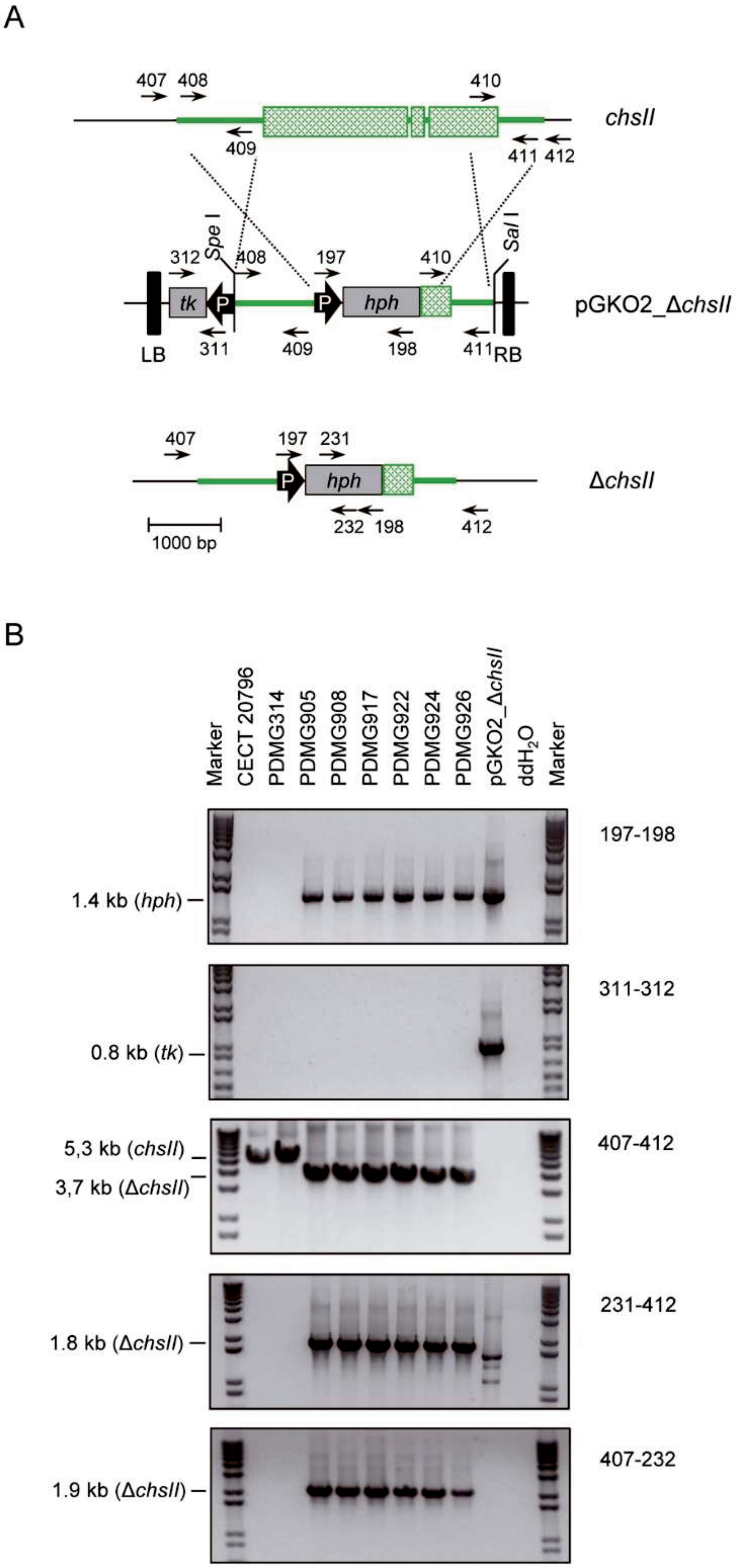
A
407
408
410
chsII
409
411
412
Spe I
Sal I
312
408
197
410
tk
P
P
hph
pGKO2_ΔchsII
LB
311
409
198
411
RB
407
197
231
P
hph
ΔchsII
232
198
412
1000 bp
B
Marker
CECT 20796
PDMG314
PDMG905
PDMG908
PDMG917
PDMG922
PDMG924
PDMG926
pGKO2_ΔchsII
ddH2O
Marker
197-198
1.4 kb (hph)
311-312
0.8 kb (tk)
407-412
5,3 kb (chsII)
3,7 kb (ΔchsII)
231-412
1.8 kb (ΔchsII)
407-232
1.9 kb (ΔchsII)

Supplementary Figure S1. Generation of *P. digitatum chsII* deletion strains and confirmation by PCR analysis. (A) Schematic representation of the *chsII* gene in CECT 20796 and PDMG314 strains, the pGKO2_Δ*chsII* vector designed for gene deletion and the Δ*chsII* deleted gene. All primers used for PCR analysis are localized in the figure. (B) PCR amplification of genomic DNA of the distinct *P. digitatum* strains with different primer pairs as indicated. In the first panel, all transformants showed an amplicon with primers OJM197/OJM198 indicating the presence of the hygromycin marker. The second panel showed a positive fragment of 800 bp, using primers OJM311/OJM312 for HSV*tk* gene only in the pGKO2_Δ*chsII* vector, as expected. The third panel displays PCR amplification with primers OJM407/OJM412. Amplicons of 3.7 kb were obtained with deletion mutants (PDMG905 to PDMG926) while parental strains CECT 20796 and PDMG314 showed higher amplicon of 5.3 kb. Using primers OJM231/OJM412 from the 3´region of the deleted *chsII* gene, the homologous recombinants produced an amplification product of 1.8 kb while no amplicon was detected in the parental strains with this primer combination (fourth panel). Similarly, primer combination OJM407/OJM232 from the 5´region showed presence of amplification products of 1.9 kb in the deletion strains (PDMG905 to PDMG926), and no amplicon in CECT 20796 or PDMG314 (fifth panel).

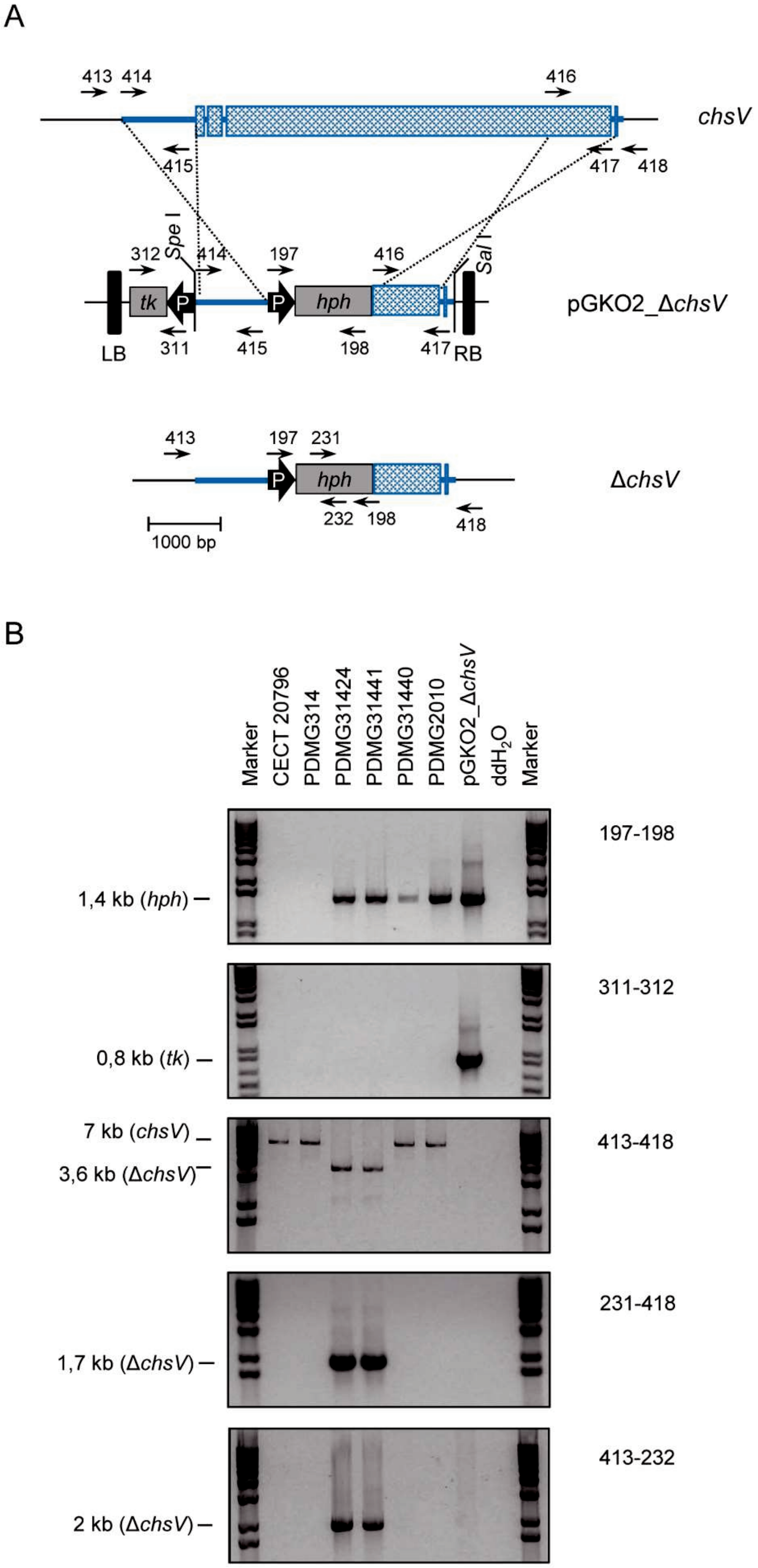
A
413
414
416
chsV
415
417
418
312
Spe I
414
197
416
Sal I
tk
P
P
hph
pGKO2_ΔchsV
LB
311
415
198
417
RB
413
197
231
P
hph
ΔchsV
232
198
418
1000 bp
B
Marker
CECT 20796
PDMG314
PDMG31424
PDMG31441
PDMG31440
PDMG2010
pGKO2_ΔchsV
ddH2O
Marker
197-198
1,4 kb (hph)
311-312
0,8 kb (tk)
7 kb (chsV)
3,6 kb (ΔchsV)
413-418
231-418
1,7 kb (ΔchsV)
413-232
2 kb (ΔchsV)

Supplementary Figure S2. Generation of *P. digitatum chsV* deletion strains and confirmation by PCR analysis. (A) Schematic representation of the *chsV* gene in wild type CECT 20796 and parental strain PDMG314, the pGKO2_Δ*chsV* vector designed for gene deletion and the Δ*chsV* deleted gene. All primers used for PCR analysis are localized in the figure. (B) PCR amplification of genomic DNA of the distinct *P. digitatum* strains with different primer pairs as indicated. In the first panel all the transformants showed an amplicon with primers OJM197/OJM198 indicating the presence of the hygromycin marker. The second panel shows a positive fragment of 800 bp, using primers OJM311/OJM312 for HSV*tk* gene, only in the pGKO2_Δ*chsV* vector, as expected. The third panel shows PCR amplification with primers OJM413/OJM418. Deletion mutants PDMG31424 and PDMG31441 showed amplicons of 3.6 kb indicating *chsV* gene deletion while CECT 20796, parental strain PDMG314 and ectopic controls (PDMG31440 and PDMG2010) showed higher amplicon of 7 kb. Using primers OJM231/OJM418 from the 3´region of the deleted *chsV* gene, the homologous recombinants produced an amplification product of 1.7 kb while no amplicon was detected in the wild type, parental and ectopic strains with this primer combination (fourth panel). Similarly, primer combination OJM413/OJM232 from the 5´region, showed presence of amplification products of 2 kb in the deletion strains and no amplicon in wild type, parental or ectopic strains (fifth panel).

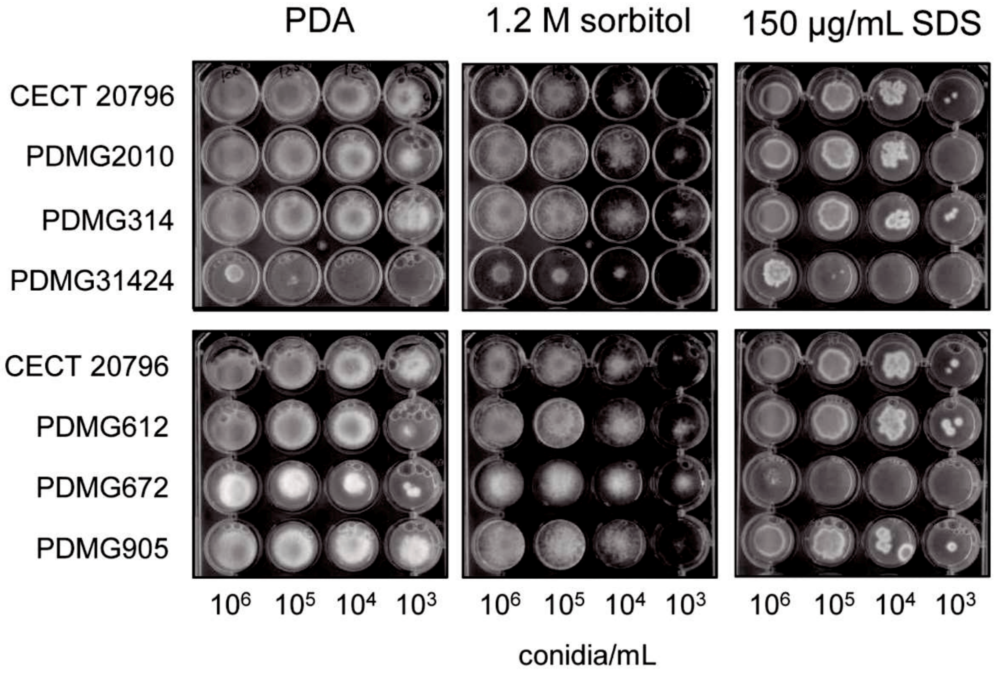

Supplementary Figure S3. Sensitivity of *P. digitatum* strains to different compounds. Comparison of different strains growth on PDA plates and PDA supplemented with indicated compounds. Serial 1/10 dilutions of conidia of each strain were applied as indicated. Different strains were parental strains CECT 20796 and PDMG314; deletion strains Δ*chsII* (PDMG905); Δ*chsV* (PDMG31424) and Δ*chsVII* (PDMG672) and two different ectopic strains PDMG2010 (*chsV*) and PDMG612 (*chsVII*).

1.1 Supplementary Tables

Supplementary Table S1. Primers used for fungal transformation and verification of transformant strains.

Name	Use	Sequence 5´- 3´	Tm (ºC)	Restriction sites	Gene
OJM407	F	GGGCGAACGATGACGAGAGGC	60		*ChsII*
OJM408	F	CA**ACTAGT**TTTGATCAAGTGGCGCACTCATGTG	60	*Spe*I	*ChsII*
OJM409	R	ATGCTCCTTCAATATCAGTTAACGGGGCCCATGTGGAGGTAGAGTGG	60		*ChsII*
OJM410	F	CCGACCGGGAACCAGTTAACACCGCTTATGCCATCTTGAACG	60		*ChsII*
OJM411	R	CA**GTCGAC**CGGTGAGCAGGTTGGACTTGGGGC	60	*Sal*I	*ChsII*
OJM412	R	GGAAATCCCCTCTGATACCCTGCC	60		*ChsII*
OJM413	F	CCTGTAGAATGATAGATGGGG	60		*ChsV*
OJM414	F	GG**ACTAGT**TTCGCCACGCAATGTATGATCGG	60	*SpeI*	*ChsV*
OJM415	R	ATGCTCCTTCAATATCAGTTAACGCGGGACGACAAGACTCAACGG	60		*ChsV*
OJM416	F	CCGACCGGGAACCAGTTAACACCGTTGTCATGCTTGGACAGTTGC	60		*ChsV*
OJM417	R	CA**GTCGAC**CACACAGAGAGTTGAATGGTGTAG	60	*Sal*I	*ChsV*
OJM418	R	CTCATCCATGCATAAGTTCGC	60		*ChsV*
OJM197	F	C**GTTAAC**TGATATTGAAGGAGCAT	60	*Hpa*I	P*trpC*
OJM198	R	T**GTTAAC**TGGTTCCCGGTCGG	60	*Hpa*I	*hph*
OJM231	F	GTTGCAAGACCTGCCTGAAACC	60		*hph*
OJM232	R	GTTTGCCAGTGATACACATGGG	60		*hph*
OJM311	F	CCACGGAAGTCCGCCCGGAGC	60		HSV*tk*
OJM312	R	GACGTGCATGGAACGGAGGCG	60		HSV*tk*

Complementary sequences are underlined and restriction sites are in bold.
